# Reply to Comment on “Osteopontin, Macrophage Migration Inhibitory Factor and Anti-Interleukin-8 Autoantibodies Complement CA125 for Detection of Early Stage Ovarian Cancer” *Cancers* 2019, *11*, 596: Markers for Early Detection of Ovarian Cancer

**DOI:** 10.3390/cancers11091386

**Published:** 2019-09-18

**Authors:** Jing Guo, Zhen Lu, Robert C. Bast

**Affiliations:** 1 Department of Obstetrics and Gynecology, Shanghai Tenth People’s Hospital, Tongji University School of Medicine, Shanghai 200072, China; 2 Department of Experimental Therapeutics, University of Texas M.D. Anderson Cancer Center, Houston, TX 77030, USA

We appreciate Mor et al. [1] correcting our inadvertent oversight of their 2005 paper [2] regarding osteopontin and macrophage inhibitory factor (MIF) as serum biomarkers for ovarian cancer. We had cited their report regarding MIF expression in ovarian cancer by Agarwal and Mor et al in 2007 [3]. We had also cited the first reports regarding osteopontin in serum of ovarian cancer patients by Schorge et al. [4] and in CA125 negative ovarian cancer tissue by Rosen et al. [5]. 

As we had noted in the manuscript, the novelty of our report deals not with the discovery of new biomarkers for ovarian cancer, but rather their evaluation in large numbers of cases with early stage (I–II) disease. In our study, sera from 76 early stage ovarian cancer patients were tested in the discovery set and from 71 early stage patients in an independent validation set, totaling 147, which is several fold more than in previous reports.

We agree that osteopontin and possibly MIF deserve further evaluation as part of biomarker panels for detection of early stage ovarian cancer.
